# A General Quantitative Genetic Model for Haplotyping a Complex Trait in Humans

**DOI:** 10.2174/138920207782446179

**Published:** 2007-08

**Authors:** Song Wu, Jie Yang, Chenguang Wang, Rongling Wu

**Affiliations:** Department of Statistics, University of Florida, Gainesville, FL 32611, USA

**Keywords:** Complex trait, diplotype, haplotype, quantitative genetics, quantitative trait nucleotides.

## Abstract

Uncertainty about linkage phases of multiple single nucleotide polymorphisms (SNPs) in heterozygous diploids challenges the identification of specific DNA sequence variants that encode a complex trait. A statistical technique implemented with the EM algorithm has been developed to infer the effects of SNP haplotypes from genotypic data by assuming that one haplotype (called the risk haplotype) performs differently from the rest (called the non-risk haplotype). This assumption simplifies the definition and estimation of genotypic values of diplotypes for a complex trait, but will reduce the power to detect the risk haplotype when non-risk haplotypes contain substantial diversity. In this article, we incorporate general quantitative genetic theory to specify the differentiation of different haplotypes in terms of their genetic control of a complex trait. A model selection procedure is deployed to test the best number and combination of risk haplotypes, thus providing a precise and powerful test of genetic determination in association studies. Our method is derived on the maximum likelihood theory and has been shown through simulation studies to be powerful for the characterization of the genetic architecture of complex quantitative traits.

## INTRODUCTION 

The high-throughout technology of single nucleotide polymorphisms (SNPs) provides a powerful tool for studying the detailed genetic and developmental architecture of complex traits, such as human diseases, because SNPs residing within a coding sequence can alter the biological function of a protein that forms a phenotype [1-2]. However, current experimental techniques have still not achieved a point at which multiple SNPs can be easily observed at their diplotype level [3]. Such a technological limitation makes it difficult to associate the phenotypes of a trait with specific DNA sequence variants (known as haplotypes) constructed by a set of SNPs, although recent genetic studies suggest that a gene may determine a complex trait through its haplotype rather than genotype [4-8]. More recently, a statistical model has been derived to estimate and test haplotype effects on trait variation with a random sample drawn from a natural population [9-11]. This model implements the population genetic properties of gene segregation into a unifying mixture-model framework for haplotype discovery. It assumes that one haplotype composed of alleles at multiple SNPs is different from the remaining haplotypes in terms of genetic effects on a trait. The former is called the reference or risk haplotype [9], whereas the latter is collectively called the non-reference or non-risk haplotype. This simplified assumption allows the direct use of a traditional biallelic quantitative genetic model [12] and facilitates the definition and estimation of genetic effects triggered by different haplotypes, but it is limited in practical use when there is substantial variation among the non-risk haplotypes. 

The motivation of this work is to expand Liu *et al*.’s [9] original idea to model all possible effects of individual haplotypes by constructing a multi-allelic quantitative genetic model within the mixture model framework. The multi-allelic model deals with genetic effects triggered by multiple alleles at a single gene and is thought to be important for explaining genetic variation in a natural population. We use the multi-allelic model to define various additive and dominance effects due to multiple risk haplotypes. Conventional model selection criteria are incorporated to choose the optimal number and combination of risk haplotypes responsible for quantitative variation of a trait. We derived closed forms for the EM algorithm to estimate a variety of genetic parameters including haplotype frequencies and haplotype effects. Simulation studies are used to test the statistical behavior of the model and validate its usefulness and utilization. 

## METHOD

### Population and Quantitative Genetic Models 

Suppose there are genetically associated SNPs each with two alleles designated as 1 and 0. Let *p* and *q* be the 1-allele frequencies for the first and second SNP, respectively. Thus, the 0-allele frequencies at different SNPs will be 1-*p* and 1-*q*. These two SNPs segregating in a natural population form four haplotypes, 11, 10, 01 and 00, whose frequencies are constructed by allele frequencies and linkage disequilibrium (*D*) between the two SNPs, i.e., *p*_11_ = *pq* + *D*, *p*_10_ = p(1 – *q*) – *D*, *p*_01_ = (1 – *p*)*q* – *D*, and *p*_00_ = (1 – *p*)(1 – *q*) – *D*. We use Θ_*p*_ = (*p*_11_, *p*_10_, *p*_01_, *p*_00_) to denote the haplotype frequency vector. These haplotypes unite randomly to generate 10 distinct diplotypes and 9 distinct genotypes. If the population is at Hardy-Weinberg equilibrium, the frequency of a diplotype is expressed as the product of the frequencies of the two haplotypes that constitute the diplotype (Table **1**). The frequency of the double zygotic genotype is the summation of the frequencies of its two possible diplotypes. 

We are interested in the detection of risk haplotype(s) constructed by the alleles of the two SNPs which encodes a quantitative trait. Below given are different genetic models used to identify risk haplotypes. 

### Biallelic Model

Liu *et al*. [9] assumed that all haplotypes are sorted into two groups, risk and non-risk, and defined the combination of risk and non-risk haplotypes as a composite diplotype. Let  *A*_1_ and *A*_0_ be the risk and non-risk haplotypes, respectively, which are equivalent to two alternative alleles if the two associated SNPs considered are viewed as a “locus”. Thus, for such a “biallelic locus”, we have three possible composite diplotypes whose genotypic values are specified as 

**(1):** 

Composite Diplotype	Genotypic Value
*A*_1_*A*_1_	*µ*_1_ = *µ* + *a*
*A*_1_*A*_0_	* µ*_2_ = *µ* + *d*
*A*_0_* A*_0_	*µ*_3_ = *µ* - *a*

where  *μ* is the overall mean, *a* is the additive effect due to the substitution of the risk haplotype by the non-risk haplotype, and *d* is the dominance effect due to the interaction between the risk and non-risk haplotypes. These parameters are arrayed in Θ_*qB*_ = (*μ, a, d*). 

There are a total of seven options to choose the risk haplotype. First, because any one haplotype from 11, 10, 01 and 00 can be risk, there are four choices for determining the risk haplotype. Second, any two haplotypes can be different from the rest, which includes three possibilities for combining the risk vs. non-risk haplotypes. All these options can be tabulated as follows: 

**(2):** 

No.	Risk Haplotype	Non-risk Haplotype
*B_1_*	11	10,01,00
*B_2_*	10	11,01,00
*B_3_*	01	11,10,00
*B_4_*	00	11,10,01
*B_5_*	11,10	01,00
*B_6_*	11,01	10,00
*B_7_*	11,00	10,01

The optimal choice of a risk haplotype for the biallelic model is based on the maximum of the likelihoods calculated for each of the seven options described above. 

### Triallelic Model

It is possible that there are two distinct risk haplotypes which are each different from non-risk haplotypes. This case is regarded as a “triallelic locus". Let *A*_1_ and *A*_2_ be the first and second risk haplotypes, and *A*_0_ be the non-risk haplotype, which form six composite diplotypes with genotypic values expressed as 

**(3):** 

Composite Diplotype	Genotypic Value
*A*_1_*A*_1_	*µ*_1_ = *µ* + *a*_1_
*A*_2_*A*_2_	*µ*_2_ = *µ* + *a*_2_
*A*_0_*A*_0_	*µ*_3_ = *µ* - *a*_1_ - *a*_2_
*A*_1_*A*_2_	$\mu_{4}=\mu +\frac{1}{2}\left(a_{1}+a_{2}\right)+d_{12}$
*A*_1_*A*_0_	$\mu_{5}=\mu -\frac{1}{2}a_{1}+d_{10}$
*A*_2_*A*_0_	$\mu_{6}=\mu -\frac{1}{2}a_{1}+d_{20}$

where *μ* is the overall mean, *a*_1_ and  *a*_2_ are the additive effects due to the substitution of the first and second risk haplotype by the non-risk haplotype, and *d*_12_, *d*_10_ and *d*_20_ are the dominance effects due to the interaction between the first and second risk haplotype, between the first risk haplotype and the non-risk haplotype and between the second risk haplotype and non-risk haplotype, respectively. These parameters are arrayed in Θ_*qT*_ = (*μ*,* a*_2_, *a*_2_, *d*_12_, *d*_10_, *d*_20_).

The triallelic model may include a total of six haplotype combinations, which are 

**(4):** 

No.	Risk Haplotype		Non-risk Haplotype
	1	2	
*T1*	11	10	01,00
*T*_2_	11	01	10,00
*T*_3_	11	00	10,01
*T*_4_	10	01	11,00
*T*_5_	10	00	11,01
*T*_6_	01	00	11,10

The optimal combination of risk haplotypes for the triallelic model corresponds to the maximum of the likelihoods calculated for each of the six possibilities. 

### Quadriallelic Model

If there are three distinct risk haplotypes, we need a quadriallelic genetic model to specify haplotype effects. Let *A*_1_, *A*_2_ and *A*_3_ be the first, second and third risk haplotypes, and *A*_0_ be the non-risk haplotype, which form 10 composite diplotypes with genotypic values expressed as 

**(5):** 

Composite Diplotype	Genotypic Value
*A*_1_*A*_1_	*µ*_1_ = *µ* + *a_1_*
*A*_2_*A*_2_	*µ*_2_ = *µ* + *a_2_*
*A*_3_*A*_3_	*µ*_3_ = *µ* + *a_3_*
*A*_0_*A*_0_	*µ*_4_ = *µ* - (*a_1_ + a_2_ + a_3_*)
*A*_1_*A*_2_	$\mu_{5}=\mu +\frac{1}{2}\left(a_{1}+a_{2}\right)+d_{12}$
*A*_1_*A*_3_	$\mu_{6}=\mu +\frac{1}{2}\left(a_{1}+a_{2}\right)+d_{13}$
*A*_2_*A*_3_	$\mu_{7}=\mu +\frac{1}{2}\left(a_{2}+a_{3}\right)+d_{23}$
*A*_1_*A*_0_	$\mu_{8}=\mu -\frac{1}{2}\left(a_{2}+a_{3}\right)+d_{10}$
*A*_2_*A*_0_	$\mu_{9}=\mu -\frac{1}{2}\left(a_{1}+a_{3}\right)+d_{20}$
*A*_3_*A*_0_	$\mu_{10}=\mu -\frac{1}{2}\left(a_{1}+a_{2}\right)+d_{30}$

where *μ* is the overall mean, *a*_1_, *a*_2_ and *a*_3_ are the additive effects due to the substitution of the first, second and third risk haplotype by the non-risk haplotype, and *d*_12_, *d*_13_, *d*_23_, *d*_10_, * d*_20_, and *d*_30_ are the dominance effects due to the interaction between the first and second risk haplotype, between the first and third risk haplotype, between the second and third risk haplotype, between the first risk and non-risk haplotype, between the second risk and non-risk haplotype, and between the third risk and non-risk haplotype, respectively. These parameters are arrayed in  Θ_*qQ*_ = (*μ*, *a*_1_, *a*_2_, *a*_3_ *d*_12_, * d*_13_, *d*_23_, *d*_10_, * d*_20_, *d*_30_).

## LIKELIHOOD

Assume that a total of *n* subjects are sampled from a Hardy-Weinberg equilibrium population and that each subject is genotyped for many SNPs and phenotyped for a quantitative trait. Consider two of the SNPs that form nine genotypes with observed numbers generally expressed as  *n_r__1_r'_1_/r_2_r'_2_* (*r*_1_, *r'*_1_, *r*_2_, *r'*_2_ = 1,0). The phenotypic value of the trait for subject *i* is expressed in terms of the two-SNP haplotypes as

(eq6) $$y_{i}=\sum_{j=1}^{J}\xi_{i}\mu_{J}+e_{i},$$

where *ξ_ i_* is the indicator variable defined as 1 if subject *i* has a composite diplotype *j* and 0 otherwise, *e_i_* is the residual error, normally distributed as *N*(0, σ^2^), and  *J* is the number of composite diplotypes expressed as

(eq7) $$J=\left\{\begin{array}{c} 3\text{ for the biallelic model}\\ 6\text{ for the triallelic model}\\ 10\text{ for the quadriallelic model.} \end{array}\right.$$

The genotypic values of composite diplotypes and variance are arrayed by a quantitative genetic parameter vector  Θ_*q*_ = (Θ_*qB*_, σ^2^) for the biallelic model, (Θ_*qT*_, σ^2^) for the triallelic model, and  (Θ_*qQ*_, σ^2^) for the quadriallelic model.

The log-likelihood of haplotype frequencies, genotypic values of the diplotypes and residual variance given the phenotypic (**y**) and SNP data (**S**) is factorized into two parts, expressed as

(eq8) $$\text{log }L\left(\Theta_{p},\Theta_{q}|y,S\right)=\text{log }L\left(\Theta_{p}|S\right)+\text{log }L\left(\Theta_{q}|y,S,\Theta_{p}\right)$$

where

(eq9) $$\begin{array}{c} \text{log }L\left(\Theta_{p}|S\right)=\text{constant}\\ +2n_{11/11}\text{ ln}p_{11}+n_{11/10}\text{ ln}\left(2p_{11}p_{10}\right)+2n_{11/00}\text{ ln}p_{10}+n_{10/11}\text{ ln}\left(2p_{11}p_{01}\right)+n_{10/10}\text{ ln}\left(2p_{11}p_{00}+2p_{10}p_{01}\right)+n_{10/00}\text{ ln}\left(2p_{10}p_{00}\right)+2n_{00/11}\text{ ln}p_{01}+n_{00/10}\text{ ln}\left(2p_{01}p_{00}\right)+2n_{00/00}\text{ ln}p_{00}, \end{array}$$

(eq10) $$\text{log L}\left(\Theta_{\text{qB}}|y,S,\Theta_{p}\right)=\sum_{i=1}^{n_{11/11}}\text{log}f_{1}\left(y_{i}\right)+\sum_{i=1}^{n_{11/10}}\text{log}f_{2}\left(y_{i}\right)+\sum_{i=1}^{n_{11/00}}\text{log}f_{3}\left(y_{i}\right)+\sum_{i=1}^{n_{10/11}}\text{log}f_{2}\left(y_{i}\right)+\sum_{i=1}^{n_{10/10}}\text{log}[\phi f_{2}\left(y_{i}\right)+\left(1-\phi \right)f_{3}\left(y_{i}\right)]+\sum_{i=1}^{n_{10/00}}\text{log}f_{3}\left(y_{i}\right)+\sum_{i=1}^{n_{00/11}}\text{log}f_{3}\left(y_{i}\right)+\sum_{i=1}^{n_{00/10}}\text{log}f_{3}\left(y_{i}\right)+\sum_{i=1}^{n_{00/00}}\text{log}f_{3}\left(y_{i}\right)$$

for the biallelic model assuming that haplotype 11 is a risk haplotype,

(eq11) $$\text{log}L\left(\Theta_{\text{qT}}|y,S,\Theta_{p}\right)=\sum_{i=1}^{n_{11/11}}\text{log}f_{1}\left(y_{i}\right)+\sum_{i=1}^{n_{11/10}}\text{log}f_{4}\left(y_{i}\right)+\sum_{i=1}^{n_{11/00}}\text{log}f_{2}\left(y_{i}\right)+\sum_{i=1}^{n_{10/11}}\text{log}f_{5}\left(y_{i}\right)+\sum_{i=1}^{n_{10/10}}\text{log}[\phi f_{5}\left(y_{i}\right)+\left(1-\phi \right)f_{6}\left(y_{i}\right)]+\sum_{i=1}^{n_{10/00}}\text{log}f_{6}\left(y_{i}\right)+\sum_{i=1}^{n_{00/11}}\text{log}f_{3}\left(y_{i}\right)+\sum_{i=1}^{n_{00/10}}\text{log}f_{3}\left(y_{i}\right)+\sum_{i=1}^{n_{00/00}}\text{log}f_{3}\left(y_{i}\right)$$

for the triallelic model assuming that haplotypes 11 and 10 are the first and second risk haplotypes, respectively,

(eq12) $$\text{log }L\left(\Theta_{\mathrm{qQ}}|y,S,\Theta_{p}\right)=\sum_{i=1}^{n_{11/11}}\text{log}f_{1}\left(y_{i}\right)+\sum_{i=1}^{n_{11/10}}\text{log}f_{5}\left(y_{i}\right)+\sum_{i=1}^{n_{11/00}}\text{log}f_{2}\left(y_{i}\right)+\sum_{i=1}^{n_{10/11}}\text{log}f_{6}\left(y_{i}\right)+\sum_{i=1}^{n_{10/10}}\text{log}[\phi f_{8}\left(y_{i}\right)+\left(1-\phi \right)f_{7}\left(y_{i}\right)]+\sum_{i=1}^{n_{10/00}}\text{log}f_{9}\left(y_{i}\right)+\sum_{i=1}^{n_{00/11}}\text{log}f_{3}\left(y_{i}\right)+\sum_{i=1}^{n_{00/10}}\text{log}f_{10}\left(y_{i}\right)+\sum_{i=1}^{n_{00/00}}\text{log}f_{4}\left(y_{i}\right)$$

for the quadriallelic model assuming that haplotypes 11, 10 and 01 are the first, second and third risk haplotypes, respectively, with *f_i_* (*y_i_*) being a normal distribution density function of composite diplotype *j* with mean *μ_j_* and variance *σ*^2^. 

We have shown that maximizing *L*(Θ_*p*_, Θ_*q*_ | **y**, **S**) in equation (8) is equivalent to individually maximizing log *L*(Θ_*p*_ | **S**) in equation (9) and log *L*(Θ_*q*_ | **y**, **S**,****Θ_*p*_) in equation (10), (11) or (12) (unpublished results). 

## THE EM ALGORITHM 

A closed-form solution for the EM algorithm has been derived to estimate the unknown parameters that maximize the likelihoods. The estimates of haplotype frequencies are based on the log-likelihood function *L*(Θ_*p*_ | **S**), whereas the estimates of genotypic values of composite diplotypes and the residual variance are based on the log-likelihood function *L*(Θ_*q*_ | **y**, **S**,****Θ_*p*_). These two different types of parameters can be estimated using a two-stage hierarchical EM algorithm (see [9] for a detailed implementation).

## MODEL SELECTION 

The formulation of likelihoods (10), (11) and (12) is based on the assumption that one or more haplotypes are risk haplotypes for the biallelic, triallelic and quadriallelic model. However, a real risk haplotype under each of these models is unknown from raw data (**y**, **S**). Also, we are uncertain about the optimal number of risk haplotypes. An additional step for the choice of the most likely risk haplotypes and their number should be implemented. The simplest way to do so is to calculate and compare the likelihood values within the model by assuming that any one or more of the four haplotypes can be a risk haplotype, and AIC or BIC among the models by assuming different numbers of risk haplotypes [13]. Thus, we obtain possible likelihood values and AIC/BIC as follows: 

**(13):** 

Model	No.	Likelihood	AIC/BIC
Biallelic	*B_l_*	$\text{log}L_{B_{l}}\left({\hat{\Theta }}_{p},{\hat{\Theta }}_{q\text{B}}|y,S\right)$	$C_{B_{l}}$
Triallelic	*T_l_*	$\text{log}L_{T_{l}}\left({\hat{\Theta }}_{p},{\hat{\Theta }}_{qT}|y,S\right)$	$C_{T_{l}}$
Quadriallelic	*Q*	$\text{log}L_{Q}\left({\hat{\Theta }}_{p},{\hat{\Theta }}_{\mathrm{qQ}}|y,S\right)$	*C_Q_*

The largest likelihood and the smallest AIC/BIC value calculated is thought to correspond to the most likely risk haplotypes and their optimal number. 

## HYPOTHESIS TESTS 

The genetic architecture of a quantitative trait is characterized by quantitative genetic parameters (including haplotype effects and the mode of their inheritance). The model proposed provides a meaningful way for estimating the genetic architecture of a trait. The estimated genotypic values for the composite diplotypes can be used to estimate additive and dominance genetic effects of haplotypes by 

**(14):** 

	Additive	Dominace
Biallelic	*a* = (*µ*_1_ - *µ*_3_)/2	*d* = *µ*_2_ - (*µ*_1_ + *µ*_3_)/2
Triallelic	*a*_1_ = [2*µ*_2_ - (*µ*_1_ + *µ*_3_)]/3	*d*_12_ = *µ*_4_ - (*µ*_1_ + *µ*_3_)/2
	*a*_2_ = [2*µ*_1_ - (*µ*_2_ + *µ*_3_)]/3	*d*_10_ = *µ*_5_ - (*µ*_1_ + *µ*_3_)/2
		*d*_20_ = *µ*_6_ - (*µ*_2_ + *µ*_3_)/2
Quadriallelic	*a*_1_ = [3*µ*_1_ - (*µ*_2_ + *µ*_3_ + *µ*_4_)]/4	*d*_12_ = *µ*_5_ - (*µ*_1_ + *µ*_2_)/2
	*a*_2_ = [3*µ*_2_ - (*µ*_2_ + *µ*_3_ + *µ*_4_)]/4	*d*_13_ = *µ*_6_ - (*µ*_1_ + *µ*_3_)/2
	*a*_3_ = [3*µ*_3_ - (*µ*_2_ + *µ*_3_ + *µ*_4_)]/4	*d*_23_ = *µ*_7_ - (*µ*_2_ + *µ*_3_)/2
		*d*_10_ = *µ*_8_ - (*µ*_1_ + *µ*_4_)/2
		*d*_20_ = *µ*_9_ - (*µ*_2_ + *µ*_4_)/2
		*d*_30_ = *µ*_10_ - (*µ*_3_ + *µ*_4_)/2

The additive and dominance effects under different models can be tested by formulating the null hypothesis that the effect being tested is equal. The estimates of the parameters under the null hypotheses can be obtained with the same EM algorithm derived for the alternative hypotheses but with a constraint of the tested effect equal to zero. The log-likelihood ratio test statistics for each hypothesis is thought to asymptotically follow a *x*^2^-distributed with the degree of freedom equal to the difference of the numbers of the parameters being tested under the null and alternative hypotheses. 

## HAPLOTYPING WITH THREE SNPS 

Li *et al*. [11] constructed a conceptual framework and statistical algorithm for haplotyping a quantitative trait with three SNPs. For a set of three SNPs, there are eight different haplotypes, among which it is possible to have one to seven risk haplotypes. The biallelic model specifies one risk haplotype which may be composed of one (8 cases), two (24 cases), three (56) or four haplotypes (170). The triallelic, quadrialleli, pentaallelic, hexaallelic, septemallelic and octoallelic models contains 28, 56, 170, 56, 24 and 8 cases, respectively. It can be seen that the model selection procedure to determine the optimal number and combination of risk haplotypes will become exponentially more complicated when the number of SNPs increases.

## MONTE CARLO SIMULATION 

The statistical properties of the model are investigated through simulation studies. Given a certain sample size of subjects (n = 100, 400 or 1000), two SNPs (each with two alleles 1 and 0) were simulated by assuming that 10 diplotypes follow a multinomial distribution with the frequencies determined by allele frequencies  *p* = 0.6 and *q* = 0.6  and linkage disequilibrium *D* = 0.05. By hypothesizing risk haplotypes under biallelic, triallelic and quadriallelic models, composite diplotypes can be determined for each double-SNP genotype. The phenotypic values of a quantitative trait were simulated as a normal distribution with mean depending on composite diplotypes and variance determined under different heritability levels (*H*^2^ = 0.1, 0.2 and 0.4).

For a practical data set, the number and combination of risk haplotypes that govern a phenotypic trait is unknown. Thus, the simulation performed here will elucidate the procedure and power to determine risk haplotypes by the new model. The data sets simulated with given risk haplotypes under each quantitative genetic model were analyzed by biallelic, triallelic and quadriallelic models, respectively. For each analysis, the likelihood and model selection criteria, AIC and BIC, are calculated with display (13). The power to correctly identify risk haplotypes was calculated from 1000 simulation replicates. Fig. (**1**) illustrates such power under different heritabilities, sample sizes and genetic models. For the data simulated under the biallelic model, a correct risk haplotype can well be determined with a sample size of 200 even when the heritability of the trait is modest (0.1). In this case in which a small number of genetic parameters are included, the BIC performs better than the AIC. For a data set simulated under the triallelic model, the power of haplotype detection reduces considerably, compared with the data set simulated by the biallelic model. If the heritability of a trait is as low as 0.1, about 1000 subjects are needed to achieve the power of 0.8. With the heritability increasing to 0.2 or 0.4, the same power needs about 600 or 300 subjects, respectively. It is interesting to note that the AIC performs better than the BIC when the heritability is low (0.1 or 0.2), whereas the two criteria perform similarly when the heritability is high (0.4). 

**Fig. (1):**
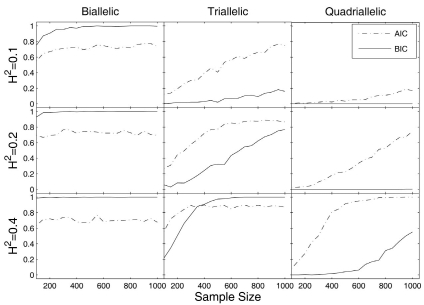
Power to detect correct risk haplotypes from the data simulated by a biallelic, triallelic and quadriallelic model, respectively, under different heritabilities and sample sizes. Model selection criteria are based on AIC and BIC.

The data set simulated under the quadriallelic model contains a very large number of genetic parameters to be estimated. As expected, the power of haplotype detection in this case will be reduced (Fig. **1**). When the heritability is as low as 0.1, a sample size of 1000 can only achieve a power of 0.2. But with an increasing heritability, the power will increase dramatically. For example, a power of > 0.9 can be achieved with 600 subjects when the heritability is 0.4. For the quadriallelic model-simulated data, the AIC always performs better than the BIC because the latter poses too heavy penalty in this case. 

The estimates of population (including allele frequencies and linkage disequilibrium) and quantitative genetic parameters (including additive and dominance effects) for each simulated data set were evaluated by calculating their sampling errors. Previous work suggested that the estimates of population genetic parameters display great precision even for a sample size of 100 [9]. Here, our simulation studies will focus on the assessment of the precision of quantitative genetic parameter estimates under different heritabilities and sample sizes. For the data set simulated with the biallelic model, the additive and dominance effects can be precisely estimated even with a heritability of 0.1 and a sample size of 100 (Table **2**). Increasing heritabilities and sample sizes increase estimation precision dramatically. 

The data set simulated under the triallelic model contains two additive effects and three dominance effects. A sample size of 100 is adequate for precise estimates of the additive effects even for a low heritability (0.1), but the reasonable estimates of the dominance effects need increasing sample size (400 or more) if the heritability is 0.1 (Table **3**). For a high heritability (0.4), a small sample size (100) can provide relatively precise estimates of the dominance effects. For the data set simulated with the quadriallelic model, three additive effects and six dominance effects are included. Still, a low sample size (100) can provide very good estimates of the additive effects even for a low heritability. To reasonably estimate the dominance effects, we need a large sample size (1000) for the heritability of 0.1 or a moderately large sample size (400) for the heritability of 0.4 (Table **4**). 

## DISCUSSION 

Single nucleotide polymorphisms (SNPs) are powerful markers that can explain interindividual differences in disease risk and drug responsiveness in humans. For genes containing multiple SNPs, haplotype structure (i.e., the linear arrangement of different SNP alleles on each of the two homologous chromosomes) is thought to be the principal determinant of phenotypic traits. While traditional analyses associate phenotypic variability with genotypes, growing evidence shows the important contribution of haplotype diversity to quantitative traits [4-8]. More recently, Liu *et al*. [9] proposed a statistical method for detecting functional (or risk) haplotypes for quantitative traits with a random sample drawn from a natural population. The method allows the characterization of DNA sequence variants that encode the phenotypic value of a trait, thus open a gateway for precisely studying the genetic architecture of quantitativevariation. In this article, we extends Liu *et al*.’s model to estimate the number and combination of multiple functional haplotypes in terms of their genetic effects.

Similar to Liu *et al*.’s work [9], our model was founded on the mixture model-based framework in which the frequencies of haplotype distribution and haplotype effects are estimated with the closed form of the EM algorithm. But our model was incorporated by two important theories from different fields, one regarding the segregation and inheritance of multiple alleles at a single locus in quantitative genetics and the second regarding model selection procedures in statistics. Liu *et al*.’s [9] model was framed on a biallelic model in which one haplotype constructed by a set of associated SNPs was assumed to perform differently from the rest of the haplotypes. Traditional quantitative genetic theory mostly based on biallelic inheritance provide a basis for estimating the additive and dominance effects due to two alternative alleles at a functional gene, but fails to characterize the genetic effects due to all possible combinations between multiple alleles. We have for the first time implemented multiallelic quantitative genetic theory into the estimation process of haplotype effects, in which multiple additive effects and multiple dominance effects due to multiple functional haplotypes can be estimated and tested separately or jointly. The new model expands the idea of haplotyping a complex trait to study the detailed genetic control of the trait in a precise way. 

To deal with multiple risk haplotypes, an issue arises naturally about the selection of most likely risk haplotypes from a pool of haplotypes. This will include the optimal number of risk haplotypes and their combination that provide a best fit to the given data. We implemented model selection procedures into the test process of haplotype diversity and effects with two commonly used criteria, AIC and BIC. Extensive simulation studies were performed to investigate the statistical properties of the model and its utilization. Given a real data set, we do not know about the type and number of risk haplotypes. But these can be estimated with model selection by assuming different types of genetic models, biallelic (one risk haplotype), triallelic (two risk haplotypes) and quadriallelic (three risk haplotypes). Simulation studies with two-SNP haplotypes provide a table of model selection approaches (Tables **2-4**) to detect most likely risk haplotypes hidden in a genetic association data set based on a range of sample size and heritability as well as the types of genetic models. 

The human genome contains millions of SNPs distributed over 23 pairs of chromosomes [14]. However, these SNPs were observed to locate in different haplotype blocks of the human genome [15-16]. For a given block, there are a particular number of representative SNPs or htSNPs that uniquely identify the common haplotypes in this block or QTN. Several algorithms have been developed to identify a minimal subset of htSNPs that can characterize the most common haplotypes [2, 17-18]. The idea given in this article can be used to find risk haplotypes of these htSNPs by modeling an arbitrary number of SNPs [11], and extended to detect haplotype-haplotype interactions [10], haplotype-environment interactions, parent-of-origin effects of haplotypes in genetic association studies and haplotypes regulating pharmacodynamic reactions of drugs [19]. Although these works will be computationally expensive, it should not be computationally prohibitive if combinatorial mathematics, graphical models, and machine learning are incorporated into closed forms of parameter estimation. With detailed extensions that take account into more realistic biological and genetic problems, our model may provide an efficient solution to the growing need for haplotype data collection and association studies.

## Figures and Tables

**Table 1.** Diplotypes and their Frequencies for each of Nine Genotypes at Two SNPs, Haplotype Composition Frequencies for Each Genotype, and Composite Diplotypes under Biallelic, Triallelic and Quadriallelic Models

Genotype	Diplotype		Relative Diplotype Frequency	Composite Diplotype		
	Configuration	Frequency		Biallelic	Triallelic	Quadriallelic
11/11	[11][11]	$p_{11}^{2}$	1	*A*_1_*A*_1_	*A*_1_*A*_1_	*A*_1_*A*_1_
11/10	[11][10]	2*p*_11_*p*_10_	1	*A*_1_*A*_0_	*A*_1_*A*_2_	*A*_1_*A*_2_
11/00	[10][10]	$p_{10}^{2}$	1	*A*_0_*A*_0_	*A*_2_*A*_2_	*A*_2_*A*_2_
10/11	[11][01]	2*p*_11_*p*_01_	1	*A*_1_*A*_0_	*A*_1_*A*_0_	*A*_1_*A*_3_
10/10	$\left\{\begin{array}{c} \left[11\right]\left[00\right]\\ \left[10\right]\left[01\right] \end{array}\right.$	$\left\{\begin{array}{c} {2p}_{11}p_{00}\\ {2p}_{10}p_{01} \end{array}\right.$	$\left\{\begin{array}{c} \phi \\ 1-\phi \end{array}\right.$	$\left\{\begin{array}{c} A_{1}A_{0}\\ A_{0}A_{0} \end{array}\right.$	$\left\{\begin{array}{c} A_{1}A_{0}\\ A_{2}A_{0} \end{array}\right.$	$\left\{\begin{array}{c} A_{1}A_{0}\\ A_{2}A_{3} \end{array}\right.$
10/00	[10][00]	2*p*_10_*p*_00_	1	*A*_0_*A*_0_	*A*_2_*A*_0_	*A*_2_*A*_0_
00/11	[01][01]	$p_{01}^{2}$	1	*A*_0_*A*_0_	*A*_0_*A*_0_	*A*_3_*A*_3_
00/10	[01][00]	2*p*_01_*p*_00_	1	*A*_0_*A*_0_	*A*_0_*A*_0_	*A*_3_*A*_0_
00/00	[00][00]	*p*^2^_00_	1	*A*_0_*A*_0_	*A*_0_*A*_0_	*A*_0_*A*_0_

Two alleles for each of the two SNPs are denoted as 1 and 0, respectively. Genotypes at different SNPs are separated by a slash. Diplotypes are the combination of two bracketed maternally and paternally derived haplotypes. Risk haplotype(s) is assumed as [11] for the biallelic model, [11] and [10] for the triallelic model, and [11], [10] and [01] for the quadriallelic model.

**Table 2.** The MLEs of the Additive and Dominance Effects Triggered by a Risk Haplotype and the Square Roots of the Mean Square Errors of the Estimates (in Parentheses) by a Biallelic Model Under Different Heritabilities and Sample Sizes

Genetic Parameter	True Value	*H*^2^ = 0.1			*H*^2^ = 0.4		
		*n* = 100	*n* = 400	*n* = 1000	*n* = 100	*n* = 400	*n* = 1000
*a*	10	10.04(0.175)	9.86(0.091)	10.04(0.055)	10.05(0.07)	10.05(0.036)	9.94(0.022)
*d*	3	2.63(0.244)	3.06(0.123)	3.11(0.08)	2.96(0.102)	2.95(0.051)	3.02(0.031)
*σ*	22.42	21.9(0.084)	22.27(0.039)	22.39(0.026)			
	9.15				9.02(0.034)	9.08(0.017)	9.13(0.011)

**Table 3.** The MLEs of the Additive and Dominance Effects Triggered by Two Risk Haplotypes and the Square Roots of the Mean Square Errors of the Estimates (in Parentheses) by a Triallelic Model Under Different Heritabilities and Sample Sizes

Genetic Parameter	True Value	*H*^2^ = 0.1			*H*^2^ = 0.4		
		*n* = 100	*n* = 400	*n* = 1000	*n* = 100	*n* = 400	*n* = 1000
*a*_1_	4.0	4.15(0.188)	4.15(0.086)	3.89(0.059)	4.04(0.076)	3.95(0.039)	4.05(0.023)
*a*_2_	-1.0	-1.24(0.192)	-1.11(0.092)	-0.84(0.057)	-0.99(0.078)	-0.95(0.039)	-1.03(0.024)
*d*_12_	-7.5	-6.91(0.582)	-7.03(0.286)	-7.52(0.169)	-7.05(0.239)	-7.67(0.114)	-7.57(0.072)
*d*_10_	-10.5	-11.26(0.409)	-10.22(0.176)	-10.55(0.121)	-10.31(0.146)	-10.47(0.075)	-10.51(0.044)
*d*_20_	-14.0	-14.25(0.288)	-13.72(0.144)	-14.1(0.091)	-13.87(0.121)	-14.06(0.058)	-14.03(0.036)
*σ*	19.11	18.43(0.07)	18.98(0.034)	19.04(0.021)			
	7.80				7.54(0.031)	7.73(0.014)	7.77(0.01)

**Table 4.** The MLEs of the Additive and Dominance Effects Triggered by Three Risk Haplotypes and the Square Roots of the Mean Square Errors of the Estimates (in Parentheses) by a Quadriallelic Model Under Different Heritabilities and Sample Sizes

Genetic Parameter	True Value	*H*^2^ = 0.1			*H*^2^ = 0.4		
		*n* = 100	*n* = 400	*n* = 1000	*n* = 100	*n* = 400	*n* = 1000
*a*_1_	-19.75	-20.91(0.815)	-19.8(0.405)	-19.88(0.266)	-20(0.346)	-19.73(0.164)	-19.76(0.105)
*a*_2_	-5.75	-2.83(0.784)	-4.48(0.393)	-5.29(0.275)	-6.35(0.344)	-6.18(0.178)	-5.93(0.117)
*a*_3_	-38.25	-37.71(0.819)	-37.97(0.354)	-38.38(0.228)	-37.94(0.307)	-38.31(0.142)	-38.21(0.093)
*d*_12_	30.00	32.99(0.47)	30.82(0.262)	29.99(0.193)	29.97(0.197)	29.97(0.118)	29.78(0.078)
*d*_13_	18.00	11.9(0.801)	15.84(0.525)	17.03(0.403)	18.55(0.358)	18.39(0.232)	18.56(0.159)
*d*_23_	23.00	23.38(0.617)	22.73(0.276)	23.09(0.174)	23.15(0.236)	23.09(0.116)	22.96(0.072)
*d*_10_	20.00	19.98(0.98)	19.9(0.433)	20.43(0.261)	19.17(0.387)	20.04(0.17)	20.11(0.113)
*d*_20_	16.00	15.91(0.625)	15.84(0.268)	15.98(0.17)	16.04(0.249)	16.03(0.116)	15.96(0.076)
*d*_30_	10.00	9.73(0.831)	9.93(0.397)	9.94(0.238)	10.06(0.363)	10.09(0.167)	9.98(0.098)
*σ*	31.50	29.48(0.12)	30.84(0.057)	31.19(0.039)			
	12.86				12.05(0.052)	12.65(0.026)	12.79(0.016)
